# Distributed source localization of epileptiform discharges in juvenile myoclonic epilepsy: Standardized low-resolution brain electromagnetic tomography (sLORETA) Study

**DOI:** 10.1097/MD.0000000000029625

**Published:** 2022-06-30

**Authors:** Kwang Yeon Kim, Ja-Un Moon, Joo-Young Lee, Tae-Hoon Eom, Young-Hoon Kim, In-Goo Lee

**Affiliations:** a Department of Pediatrics, College of Medicine, The Catholic University of Korea, Seoul, Republic of Korea.

**Keywords:** distributed source model, electroencephalography, juvenile myoclonic epilepsy, source localization, standardized low-resolution brain electromagnetic tomography

## Abstract

Juvenile myoclonic epilepsy (JME) is a common generalized epilepsy syndrome considered the prototype of idiopathic generalized epilepsy. To date, generalized and focal seizures have been the fundamental concepts for classifying seizure types. In several studies, focal features of JME have been reported predominantly in the frontal lobe. However, results in previous studies are inconsistent. Therefore, we investigated the origin of epileptiform discharges in JME.

We performed electroencephalography source localization using a distributed model with standardized low-resolution brain electromagnetic tomography. In 20 patients with JME, standardized low-resolution brain electromagnetic tomography images corresponding to the midpoint of the ascending phase and the negative peak of epileptiform discharges were obtained from a total of 362 electroencephalography epochs (181 epochs at each timepoint).

At the ascending phase, the maximal current source density was located in the frontal lobe (58.6%), followed by the parietal (26.5%) and occipital lobes (8.8%). At the negative peak, the maximal current source density was located in the frontal lobe (69.1%), followed by the parietal (11.6%) and occipital lobes (9.4%). In the ascending phase, 41.4% of discharges were located outside the frontal lobe, and 30.9% were in the negative peak. Frontal predominance of epileptiform discharges was observed; however, source localization extending to various cortical regions also was identified. This widespread pattern was more prominent in the ascending phase (*P* = .038).

The study results showed that JME includes widespread cortical regions over the frontal lobe. The current concept of generalized epilepsy and pathophysiology in JME needs further validation.

## 1. Introduction

Juvenile myoclonic epilepsy (JME) is a common generalized epilepsy syndrome that usually presents in adolescence and continues to adulthood. The clinical and electroencephalography (EEG) characteristics were reported by Janz and Christian^[1]^ early in 1957. It includes 3 seizure types: myoclonic jerks, generalized tonic-clonic seizures, and absence seizures, which are considered the prototype of idiopathic generalized epilepsy (IGE). Interictal and ictal EEGs typically show rapid (4–6 Hz), generalized spike-waves and polyspike-waves.^[2]^

To date, generalized and focal seizures have been the fundamental concepts for classifying seizure types. However, generalized seizures do not always develop equally throughout the brain.^[3]^ In 2010, the International League Against Epilepsy (ILAE) defined “generalized” as “originating at some point within, and rapidly engaging, bilaterally distributed networks.”^[4]^ The revised classification emphasized the concepts of “rapidly engaging” and “bilaterally” rather than “generalized.”

In several studies, the focal features of JME have been reported predominantly in the frontal lobe. Holmes et al^[5]^ suggested that JME arises from a network involving the frontotemporal cortex based on EEG analysis. In addition, frontal lobe dysfunction in JME has been shown in neuropsychological studies.^[6,7]^ Subsequently, studies in which multimodal methods such as conventional magnetic resonance imaging (MRI), functional MRI (fMRI), diffusion tensor imaging (DTI), and magnetoencephalography (MEG) were used have reported focal changes in JME.^[8–13]^ However, the results in these studies are inconsistent. In some studies, involvement of other regions as well as the frontal cortex was reported. In those studies, a wide range of regions, including temporal, parietal, and occipital lobes, as well as subcortical structures, were involved in JME.

EEG remains the most useful and widely used diagnostic tool in epilepsy. Recently, the source localization methods of EEG have been developed further. In source localization, the distributed source model has become much more sophisticated, with several advantages. In the distributed model, the algorithms address the inverse problem with few lead-in assumptions.^[14]^ The model is an excellent method for investigating the electrophysiological and anatomical distributions of epilepsy.^[15]^ However, only a few studies have investigated the distributed model in JME. Therefore, to better understand electrophysiological features of JME, we investigated the EEG source localization of epileptiform discharges using a distributed model.

## 2. Methods

### 2.1. Patients and EEG recordings

A total of 20 patients (9 males and 11 females), newly diagnosed with JME and not receiving anticonvulsant treatment from January 2018 to December 2020, was enrolled in this study. The diagnosis of JME was based on the ILAE classification.^[3]^ The mean age at diagnosis was 13.6 ± 2.8 years [standard deviation (SD)] and ranged from 6 to 18 years. All participants and their parents provided written informed consent before the data acquisition. Each patient underwent awake and sleeping EEG recording for 30 minutes using a NicoletOne EEG system (Natus Medical Inc.; Pleasanton, CA) with a sampling rate of 500 Hz. Activation procedures were performed with intermittent photostimulation (at 5–10–15–20–25 Hz) and hyperventilation (3 minutes). Twenty-one Ag/AgCl electrodes were placed according to the international 10 to 20 system, including the standard 16 temporal and parasagittal scalp sites along with Fz, Cz, Pz, A1, and A2. Additional electrodes for artifact identification were employed, including 2 sites near the eyes, plus recordings of respiration and electrocardiography. Nineteen-channel EEG was recorded using linked ears reference. Additional bipolar montages were used to differentiate between EEG and eye movement potentials and to detect electromyographic activity. Electrode impedance did not exceed 5 kΩ. In the EEG derivations, the filters were set at 1.0 and 70 Hz. Sixteen-bit on-line digitization was used.

### 2.2. EEG data processing and distributed source localization

Epileptiform discharges were identified by visually inspecting each EEG recording. A total of 181 epileptiform discharges was obtained from 20 patients. The number of epileptiform discharges ranged from 1 to 39 per EEG recording (mean ± SD, 9.1 ± 9.3). In each epileptiform discharge, the first discernible prominent spike of the spike-wave or polyspike-wave complex was selected. From each selected spike, 2 EEG segments were obtained within a time window of 5 ms, including the midpoint of the ascending phase and the negative peak. Thus, a total of 362 EEG segments was used for distributed source localization.

Distributed source localization was performed using standardized low-resolution brain electromagnetic tomography (sLORETA), a functional imaging method based on electrophysiological and neuroanatomical constraints.^[16]^ In sLORETA, the cortex is modeled as a collection of volume elements (6239 voxels, size 5 × 5 × 5 mm) and restricted to cortical gray matter, hippocampus, and amygdala in the digitized Montreal Neurological Institute (MNI) coordinates corrected to Talairach coordinates. In addition, neuronal activity is computed as current density (µA/mm^2^) without assuming a predefined number of active sources.^[17]^ Scalp electrode coordinates on the MNI brain are derived from the international 5% system.^[18]^ The sLORETA algorithm solves the inverse problem by assuming related orientations and strengths of neighboring neuronal sources (represented by adjacent voxels). sLORETA images corresponding to the midpoint of the ascending phase or the negative peak of the spike (181 images each) were obtained from each EEG epoch. The maximum current source density (CSD) location was considered the source origin.

## 3. Results

### 3.1. Source localization at the midpoint of the ascending phase of epileptiform discharges

**Figure 1.**
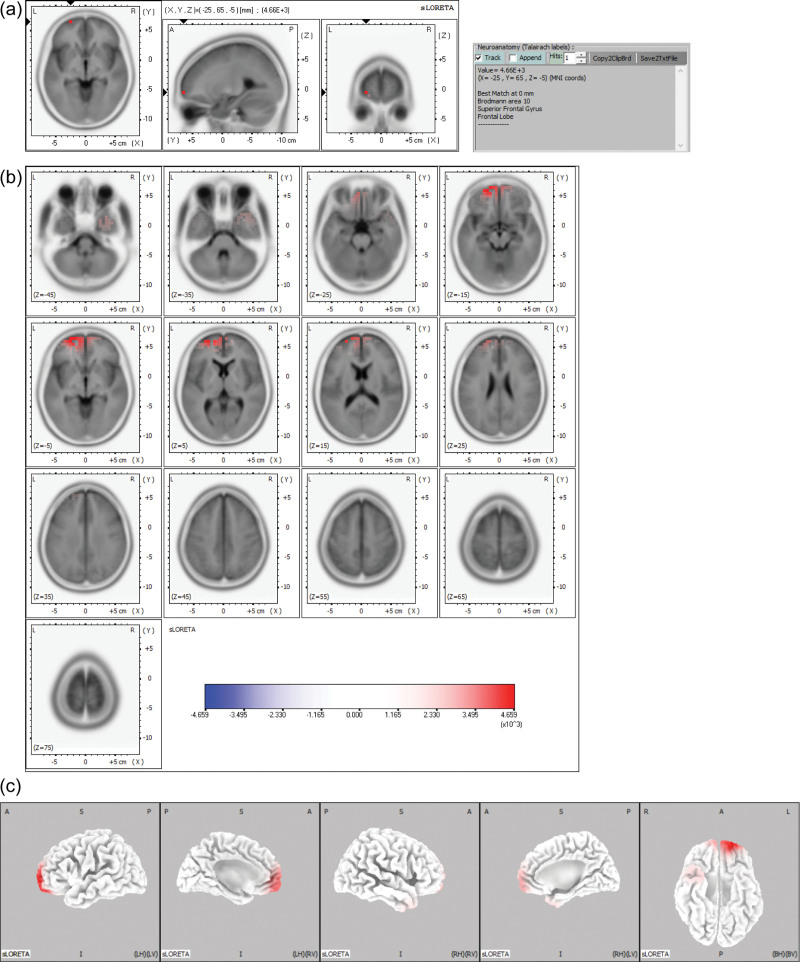
sLORETA maps of the midpoint of the ascending phase of the epileptiform discharge in Example 1 projected onto brain MRI template slices (A), (B) and three-dimensional fiducial brain cortex (C). Colored areas represent the maximal point (a) and spatial extent (B), (C) of voxels within the current source density. The color scale represents sLORETA values. A = anterior, P = posterior, S = superior I = inferior, L = left, *R* = right, B = both, H = hemisphere, V = ventricle.

**Figure 2.**
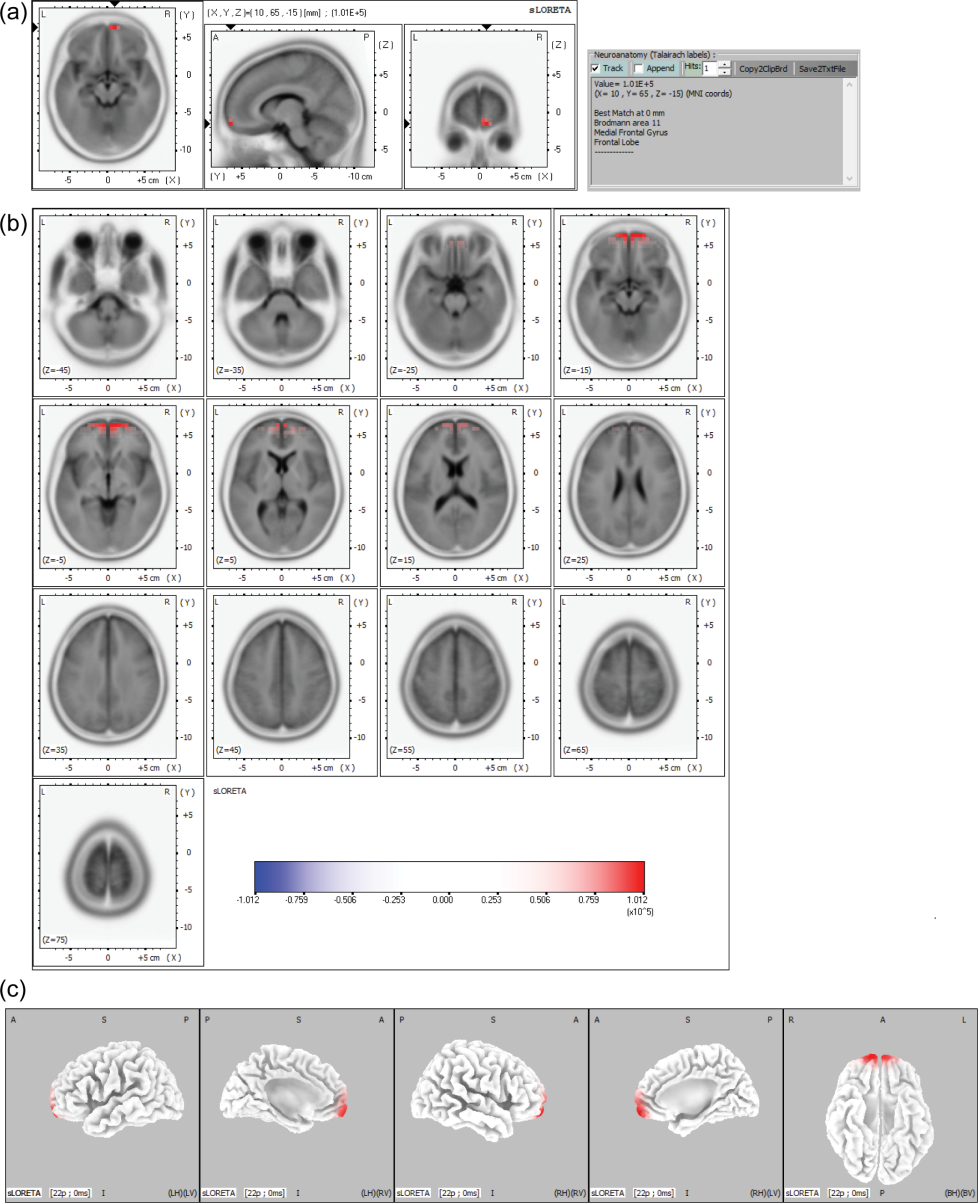
sLORETA maps of the midpoint of the ascending phase of the epileptiform discharge in Example 2 projected onto brain MRI template slices (A), (B) and three-dimensional fiducial brain cortex (C). Colored areas represent the maximal point (a) and spatial extent (B), (C) of voxels within the current source density. The color scale represents sLORETA values. A = anterior, P = posterior, S = superior I = inferior, L = left, *R* = right, B = both, H = hemisphere, V = ventricle.

The maximal CSD was most frequently located in the frontal lobe (106 discharges, 58.6%), followed by the parietal (48 discharges, 26.5%) and occipital lobes (16 discharges, 8.8%). The maximal CSD was in the limbic lobe (7 discharges, 3.9%), followed by the temporal lobe (4 discharges, 2.2%). At the gyral level of the frontal lobe, the maximal CSD was most frequent in the superior frontal gyrus (57 discharges, 31.5%), followed by the middle frontal gyrus (19 discharges, 10.5%), inferior frontal gyrus (13 discharges, 7.2%), and medial frontal gyrus (11 discharges, 6.1%). In the parietal lobe, the maximal CSD was most frequent in the postcentral gyrus (26 discharges, 14.4%), followed by the precuneus (10 discharges, 5.5%) and superior parietal lobule (9 discharges 5.0%). At the hemisphere level, the maximal CSD was located more frequently in the left hemisphere (113 discharges, 62.4%) than the right hemisphere (68 discharges, 37.6%). Table 1 summarizes the maximal CSD at the midpoint of the ascending phase of epileptiform discharges in 20 patients. Figures 1 and 2 show examples of the maximal point and the spatial extent of voxels within the CSD in the brain MRI template slices and the three-dimensional fiducial cortical surface.

**Table 1:** Location of maximal current source density at the midpoint of the ascending phase of 181 epileptiform discharges in 20 patients with juvenile myoclonic epilepsy.

Lobe		Left (n = 113)	Right (n = 68)	Total (n = 181)
Frontal (106)	Superior frontal gyrus	39	18	57
	Middle frontal gyrus	10	9	19
	Inferior frontal gyrus	4	9	13
	Medial frontal gyrus	7	4	11
	Precentral gyrus	1	0	1
	Orbital gyrus	2	0	2
	Rectal gyrus	1	1	2
	Subcallosal gyrus	1	0	1
Temporal (4)	Superior temporal gyrus	1	1	2
	Angular gyrus	2	0	2
Parietal (48)	Postcentral gyrus	20	6	26
	Superior parietal lobule	6	3	9
	Inferior parietal lobule	2	1	3
	Precuneus	3	7	10
Occipital (16)	Superior occipital gyrus	1	1	2
	Middle occipital gyrus	2	1	3
	Lingual gyrus	1	1	2
	Fusiform gyrus	2	1	3
	Cuneus	4	2	6
Limbic (7)	Anterior cingulated	2	3	5
	Posterior cingulated	1	0	1
	Uncus	1	0	1

### 3.2. Source localization at the negative peak of epileptiform discharges

**Figure 3.**
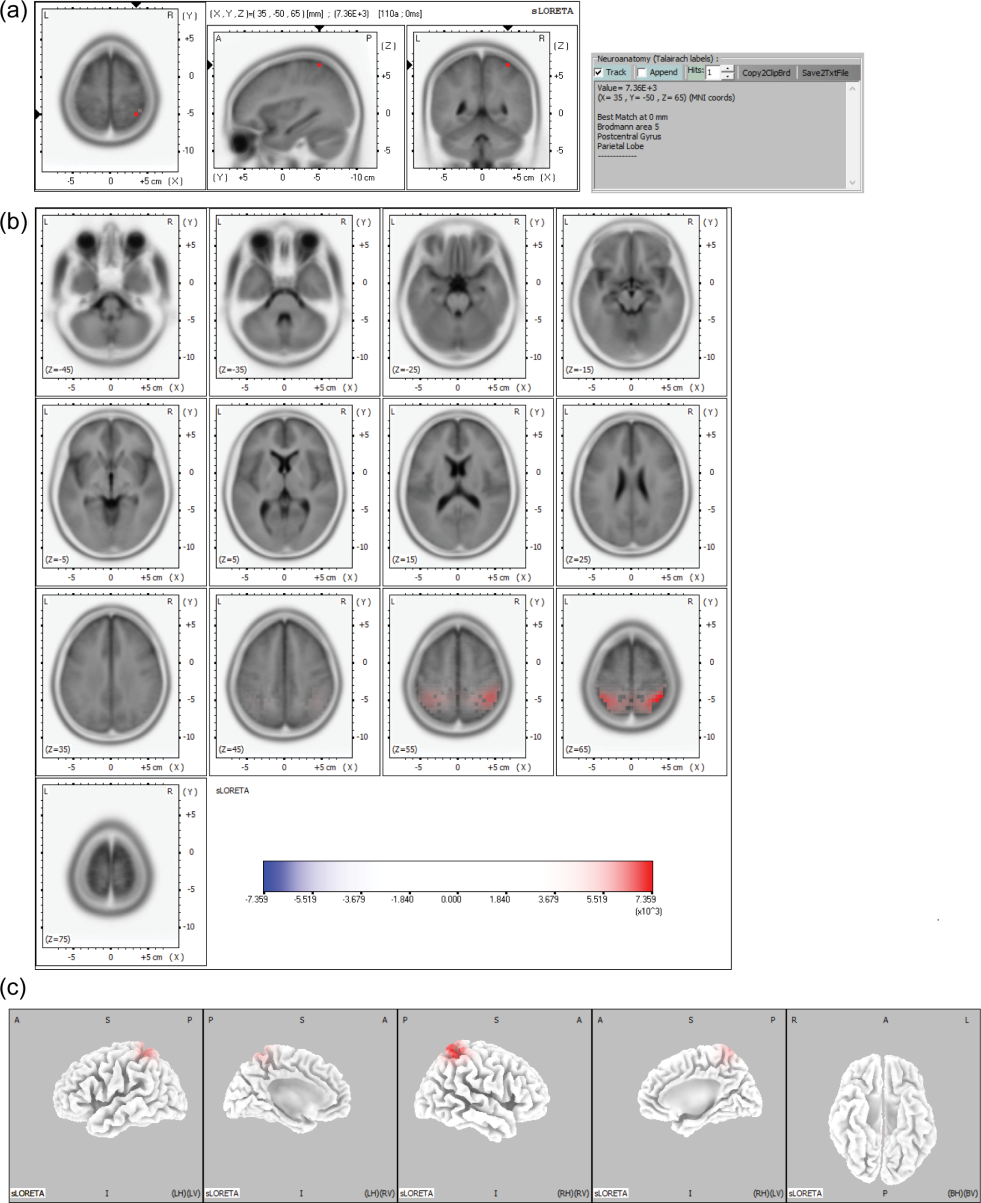
sLORETA maps of the midpoint of the ascending phase of the epileptiform discharge in Example 3 projected onto brain MRI template slices (A), (B) and three-dimensional fiducial brain cortex (C). Colored areas represent the maximal point (A) and spatial extent (B), and (C) of voxels within the current source density. The color scale represents sLORETA values. A = anterior, P = posterior, S = superior, I = inferior, L = left, *R* = right, B = both, H = hemisphere, V = ventricle.

**Figure 4.**
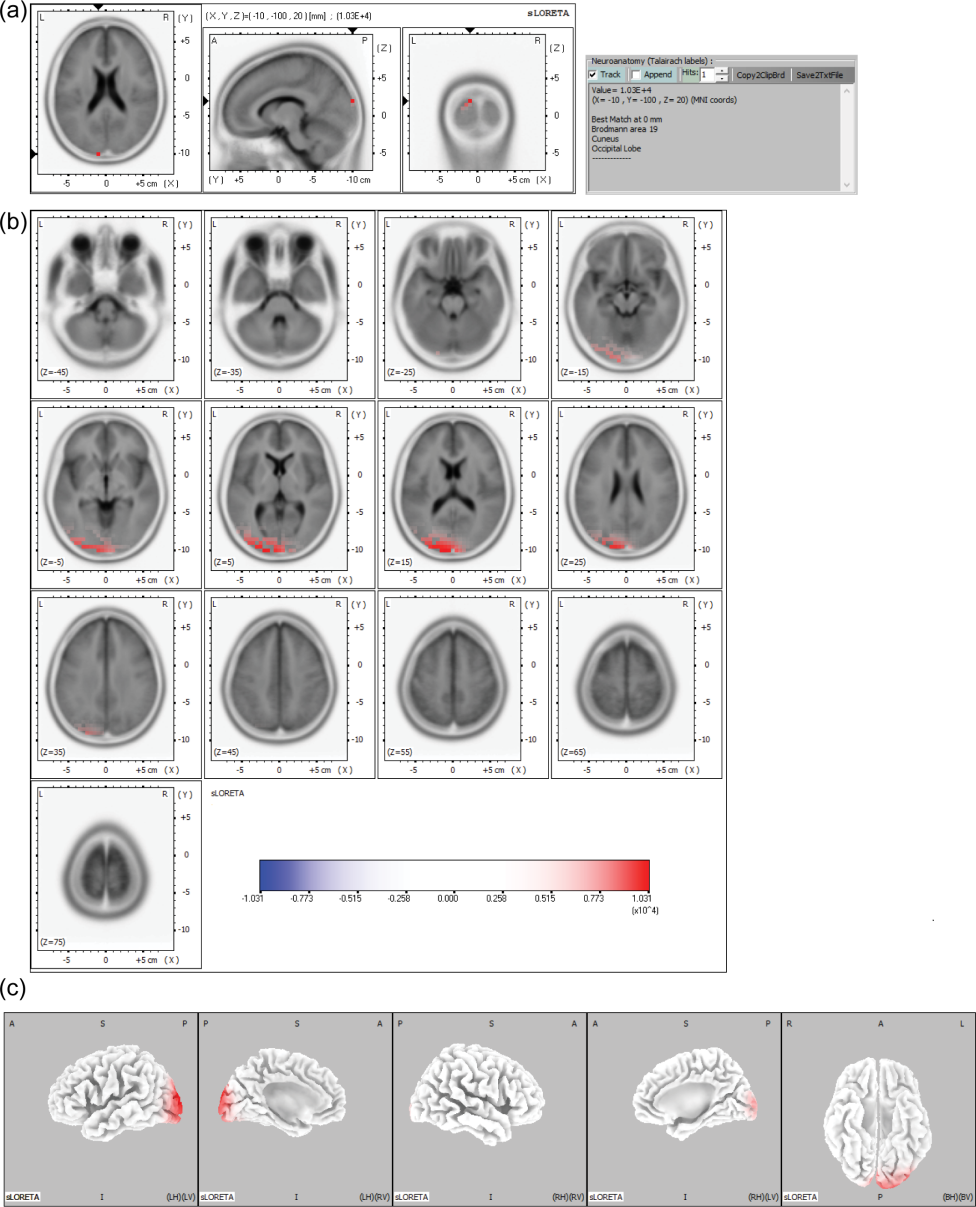
sLORETA maps of the midpoint of the ascending phase of the epileptiform discharge in Example 4 projected onto brain MRI template slices (A), (B) and three-dimensional fiducial brain cortex (C). Colored areas represent the maximal point (A) and spatial extent (B), (C) of voxels within the current source density. The color scale represents sLORETA values. A = anterior, P = posterior, S = superior, I = inferior, L = left, *R* = right, B = both, H = hemisphere, V = ventricle.

The maximal CSD was located most frequently in the frontal lobe (125 discharges, 69.1%), followed by the parietal (21 discharges, 11.6%) and occipital lobes (17 discharges, 9.4%). The maximal CSD was in the temporal lobe (11 discharges, 6.1%), followed by the limbic lobe (7 discharges, 3.9%). At the gyral level of the frontal lobe, the maximal CSD was most frequent in the superior frontal gyrus (66 discharges, 36.5%), followed by the medial frontal gyrus (27 discharges, 14.9%), middle frontal gyrus (17 discharges, 9.4%), and inferior frontal gyrus (11 discharges, 6.1%). In the parietal lobe, the maximal CSD was most frequent in the precuneus (8 discharges, 4.4%), followed by the postcentral gyrus (7 discharges 3.9%). At the hemisphere level, the maximal CSD was located more frequently in the left hemisphere (96 discharges, 53.0%) than in the right hemisphere (85 discharges, 47.0%). Table 2 summarizes the maximal CSD at the negative peak of epileptiform discharges in 20 patients. Figures 3 and 4 show examples of the maximal point and the spatial extent of voxels within the CSD in the brain MRI template slices and the three-dimensional fiducial cortical surface.

**Table 2:** Location of maximal current source density at the negative peak of 181 epileptiform discharges in 20 patients with juvenile myoclonic epilepsy.

Lobe		Left (n = 96)	Right (n = 85)	Total (n = 181)
Frontal (125)	Superior frontal gyrus	37	29	66
	Middle frontal gyrus	11	6	17
	Inferior frontal gyrus	5	6	11
	Medial frontal gyrus	12	15	27
	Orbital gyrus	1	1	2
	Rectal gyrus	1	1	2
Temporal (11)	Superior temporal gyrus	3	3	6
	Middle temporal gyrus	0	1	1
	Inferior temporal gyrus	1	0	1
	Fusiform gyrus	2	0	2
	subgyral	1	0	1
Parietal (21)	Postcentral gyrus	5	2	7
	Submarginal gyrus	1	0	1
	Superior parietal lobule	3	2	5
	Precuneus	5	3	8
Occipital (17)	Superior occipital gyrus	1	1	2
	Middle occipital gyrus	2	2	4
	Lingual gyrus	1	2	3
	Fusiform gyrus	0	2	2
	Cuneus	1	5	6
Limbic (7)	Anterior cingulate	1	4	5
	Posterior cingulate	1	0	1
	Uncus	1	0	1

## 4. Discussion

Our results showed that the frontal lobe was the most frequent source origin of epileptiform discharges in JME. In addition, at both the midpoint of ascending phase and negative peak, the source origin was located most often in the frontal lobe. If propagation of epileptiform discharges is rapid, source localization at the negative peak can reflect propagation rather than generation of epileptiform discharges.^[19]^ In several studies, the ascending phase was suggested to be more appropriate for source localization of epileptiform discharges.^[19–22]^ Therefore, we performed source localization in both ascending phase and negative peak of epileptiform discharges.

In terms of individual distribution, this frontal predominance was mostly consistent across patients in our study. At the midpoint of the ascending phase, maximal CSD was most frequently located in the frontal lobe in 18 of 20 patients. In the other 2 patients, maximal CSD was most frequent in the parietal and occipital lobes, respectively. At the negative peak, maximal CSD was most frequently located in the frontal lobe in 19 of 20 patients, and was most frequent in the parietal lobe in the other patient.

Source localization of epileptiform discharges in generalized epilepsy is of interest because generalized epilepsy refers to involvement of all parts of the brain. However, it is controversial whether generalized epilepsy is truly generalized.^[23]^ In several studies, source origin of interictal spikes in JME was reported in the frontal lobe.^[5,13,24,25]^ In neuropsychological studies, subtle cognitive deficits in JME were suggested, indicating frontal lobe dysfunction.^[6,7]^ Furthermore, in some advanced imaging studies, functional and structural abnormalities have been identified in the frontal cortex.^[8–13,26]^ The results in these studies agree with our finding of frontal predominance in source origin.

However, source localization studies in JME have been inconsistent and showed various distributions.^[13,25,27,28]^ In particular, source localization frequently involved the sensorimotor and visual cortex areas.^[25,27,28]^ These results were supported in several functional studies using EEG, fMRI, and evoked potentials.^[29–31]^ In addition, the distribution of the temporal cortex and subcortical structures, such as thalamus and cerebellum, has been reported.^[5,13,25]^

In our study, source localization of epileptiform discharges was distributed in various regions including the parietal and occipital lobes over the frontal lobe. Although focal features in generalized epilepsy have been suggested, the concept of generalized epilepsy remains valid and accepted by ILAE.^[4]^ As mentioned above, generalized epilepsy is defined as “originating at some point within, and rapidly engaging, bilaterally distributed networks.”^[3,4]^ In this context, the frontal predominance of source localization in this study can be hypothesized as a result of “more rapidly engaging” or “more highly propagated.” Although the results showed frontal predominance of the source localization, various cortical regions other than the frontal lobe were also involved. In the ascending phase, 41.4% of discharges were located outside the frontal lobe and 30.9% were also in the negative peak. Notably, this pattern was more prominent in the ascending phase (*P* = .038; chi-square test). As noted, source localization of the negative peak can reflect rapid propagation of epileptiform discharges.^[19–22]^

Furthermore, we recently published another study that investigated background EEG changes before and after anticonvulsant treatments in JME.^[32]^ In that study, focal features of EEG were observed by the source localization method, but various cortical regions including the frontal, parietal, occipital, and limbic lobes were also involved. The results were consistent with this study, suggesting that JME is distinct from focal epilepsies. It was also suggested that these distinct features might be affected by anticonvulsant treatments.

The classification of seizure types is based on operational aspects rather than pathophysiology and can be obscured by limitations of current clinical methods.^[4]^ The underlying pathophysiology of JME is not understood fully; however, the thalamocortical theory is accepted widely. Generalized spike-wave discharges are suggested to arise from abnormal oscillatory rhythm in a thalamocortical circuit.^[33,34]^ The thalamocortical networks between the thalamus and various cortical regions contribute to widespread cortical involvement.^[35,36]^ Although certain thalamocortical networks can be predominantly involved during generalized spike-wave discharges, these mechanisms are distinct from those of focal epilepsies.^[37]^

The focal features of JME have been reported by source localization studies, but few of these studies focused exclusively on JME. To the best of our knowledge, this is the first study that investigated epileptiform discharges in JME using a distributed source model. Our study results showed a frontal predominance of epileptiform discharges in JME and also confirmed the involvement of various cortical regions outside the frontal lobe. In addition, this widespread distribution was prominent in the ascending phase of the epileptiform discharges. These results indicate that JME is distinct from focal epilepsies despite its reported focal features. These features are more consistent with the concept of generalized epilepsy currently accepted by the ILAE classification.^[4]^

However, our study had several limitations. First, the study included a small number of patients, and larger sample size is needed to render the results more generalizable. In addition, analysis with sLORETA is restricted to the cortical gray matter, hippocampus, and amygdala.^[17]^ Therefore, deep subcortical structures such as the thalamus were not considered in the analysis. sLORETA is an excellent tool with no location bias; however, other neuroimaging analysis techniques also should be considered. Thus, extended studies with a larger sample size and other analysis tools are needed to confirm our findings. In addition, animal and cellular studies of the epilepsy model would elucidate the fundamental pathophysiology.

This study demonstrated that JME includes widespread cortical regions over the frontal lobe using a distributed source model. Frontal predominance of epileptiform discharges was observed; however, source localization extending to various cortical regions also was identified. The current concept of generalized epilepsy and pathophysiology in JME requires further validation. In addition, more studies are needed to further understand the characteristics and pathophysiology of JME.

### Author contributions

T.-H.E., Y.-H.K., I.-G.L. were involved conceptualization. K.-Y.K., J.-U.M., J.-Y.L., T.-H.E. were involved in data curation and formal analysis. T.-H.E. was involved in methodology and supervision and writing – review & editing. T.-H.E., Y.-H.K., and I.-G.L. were involved in resources. T.-H.E. and K.-Y.K. were involved in writing – original draft.
